# Supplementary material for: Proposal of a New Prognostic Model for Differentiated Thyroid Cancer with TERT Promoter Mutations
Source: Cancers (Basel). 2021 Jun 11;13(12):2943. doi: 10.3390/cancers13122943 (PMC8231155; doi:10.3390/cancers13122943)
Supplement: Supplementary file 1 [file cancers-13-02943-s001.zip › cancers-1231183-supplementary.pdf]

Supplementary Materials:

Proposal of a New Prognostic Model for Differentiated Thyroid Cancer with *TERT* Promoter Mutations

Jun Park, Sungjoo Lee, Jiyun Park, Hyunju Park, Chang-Seok Ki, Young Lyun Oh, Jung Hee Shin, Jee Soo Kim, Sun Wook Kim, Jae Hoon Chung, Kyunga Kim and Tae Hyuk Kim

Table S1. Regression analysis of *TERT* mutation status and clinicopathological variables in patients with DTC.

Variables	N[a]	10-year survival rate (%)	Univariate Cox models	
			HR (95% CI)	*p*-Value
Sex				
Female	329	95.1	1.00 (reference)	
Male	64	92.2	1.83 (0.78–4.34)	0.167
Age, years				
Per 5 years	393		1.75 (1.50–2.06)	<0.001
< 55	319	97.5	1.00 (reference)	<0.001
≥ 55	74	82.4	11.34 (4.96–25.93)	
TERT mutation				
Wild-type	350	98.0	1.00 (reference)	< 0.001
Mutant	43	67.4	24.15 (10.56–55.25)	
BRAF mutation				
Wild-type	117	92.3	1.00 (reference)	0.331
Mutant	199	97.0	0.64 (0.26–1.57)	
Histologic type				
PTC	327	96.6	1.00 (reference)	0.004
FTC	66	84.8	3.15 (1.44–6.88)	
Multifocality				
Absent	286	94.4	1.00 (reference)	0.847
Present	107	95.3	0.92 (0.39–2.17)	
Lymph node metastasis				
Absent	199	94.0	1.00 (reference)	0.964
Present	193	95.3	1.02 (0.47–2.20)	
Extrathyroidal extension				
Absent	352	96.0	1.00 (reference)	<0.001
Present	41	82.9	4.78 (2.14–10.64)	
Distant metastasis				
Absent	370	96.8	1.00 (reference)	<0.001
Present	23	60.9	10.85 (4.86–24.22)	
Stage at diagnosis[b]				
I	329	97.6	1.00 (reference)	<0.001
II	48	85.4	10.42 (4.19–25.92)	<0.001
III & IV	16	62.5	26.82 (10.03–71.70)	<0.001
Tumor size, cm				
<2.0	45	95.6	1.00 (reference)	0.014
2.0–4.0	293	95.9	1.24 (0.28–5.38)	0.776
>4.0	55	87.3	3.96 (0.86–18.34)	0.078

Modified form Published information: Table 3 of *Cancers*. 2020 Feb 5;13(4):648 [14].

TERT, telomerase reverse transcriptase; *BRAF*, v-Raf murine sarcoma viral oncogene homolog B; DTC, differentiated thyroid cancer; PTC, papillary thyroid cancer; FTC, follicular thyroid cancer; HR, hazard ratio. a The number based on available data for a particular variable in the univariate

analysis. b Staging according to the American Joint Committee on Cancer Thyroid Cancer Staging System 8th edition, 2016. HR are referred to the risk of thyroid cancer-specific death.

Table S2. Hazard ratios of 6 combinations of TNM-8 stage and *TERT* promoter mutation status for cancer-related death from all pairwise multivariate cox regression.

	Adjusted1*		Adjusted2*		Adjusted3*	
	**HR (95% CI)**	***p*-Value**	**HR (95% CI)**	***p*-Value**	**HR (95% CI)**	***p*-Value**
**Combinations**		<0.001		<0.001		<0.001
WT/stage I	1.00 (reference)		0.09 (0.02–0.42)	0.002	0.06 (0.01–-0.41)	0.005
WT/stage II	10.66 (2.37–47.95)	0.002	1.00 (reference)		0.60 (0.07–4.92)	0.633
WT/stage III&IV	17.82 (2.44–130.18)	0.005	1.67 (0.20–13.76)	0.633	1.00 (reference)	
MUT/stage I	20.63 (5.17–82.40)	<0.001	1.94 (0.42–8.95)	0.398	1.16 (0.16–8.28)	0.884
MUT/stage II	55.37 (16.06–190.87)	<0.001	5.19 (1.35–19.94)	0.016	3.11 (0.48–20.25)	0.236
MUT/stage III&IV	78.37 (22.44–273.67)	<0.001	7.35 (1.89–28.52)	0.004	4.40 (0.64–30.01)	0.131
	**Adjusted4***		**Adjusted5***		**Adjusted6***	
	**HR (95% CI)**	***p*-Value**	**HR (95% CI)**	***p*-Value**	**HR (95% CI)**	***p*-Value**
**Combinations**		<0.001		<0.001		<0.001
WT/stage I	0.05 (0.01–0.19)	<0.001	0.02 (0.01–0.06)	<0.001	0.01 (0.00–0.04)	<0.001
WT/stage II	0.52 (0.11–2.39)	0.398	0.19 (0.05–0.74)	0.016	0.14 (0.04–0.53)	0.004
WT/stage III&IV	0.86 (0.12–6.18)	0.884	0.32 (0.05–2.10)	0.236	0.23 (0.03–1.55)	0.131
MUT/stage I	1.00 (reference)		0.37 (0.11–1.28)	0.116	0.26 (0.07–0.94)	0.040
MUT/stage II	2.68 (0.78–9.19)	0.116	1.00 (reference)		0.71 (0.24–2.05)	0.523
MUT/stage III&IV	3.80 (1.06–13.58)	0.040	1.42 (0.49–4.11)	0.523	1.00 (reference)	

TNM-8, AJCC tumor-node-metastasis staging system 8th edition; *TERT*, telomerase reverse transcriptase; HR, hazard ratio; CI, confidence interval; WT, wild type; MUT, mutant.

* A Cox model adjusting for sex, histological type. .

Table S3. Hazard ratios of TNM-8T for cancer-related death from all pairwise multivariate cox regression.

	Adjusted1*		Adjusted2*	
	**HR (95% CI)**	***p*-Value**	**HR (95% CI)**	***p*-Value**
**Alternative grouping**		<0.001		<0.001
TNM-8T stage 1	1.00 (reference)		0.09 (0.02-0.41)	0.002
TNM-8T stage 2	10.62 (2.41-46.71)	0.002	1.00 (reference)	
TNM-8T stage 3	18.57 (5.02-68.70)	<0.001	1.75 (0.40-7.57)	0.455
TNM-8T stage 4	62.87 (20.66-191.29)	<0.001	5.92 (1.74-20.20)	0.004
	**Adjusted3***		**Adjusted4***	
	**HR (95% CI)**	***p*-Value**	**HR (95% CI)**	***p*-Value**
**Alternative grouping**		<0.001		<0.001
TNM-8T stage 1	0.05 (0.01-0.20)	<0.001	0.02 (0.01-0.05)	<0.001
TNM-8T stage 2	0.57 (0.13-2.47)	0.455	0.17 (0.05-0.58)	0.004
TNM-8T stage 3	1.00 (reference)		0.30 (0.10-0.84)	0.022
TNM-8T stage 4	3.39 (1.19-9.65)	0.022	1.00 (reference)	

HR, hazard ratio; CI, confidence interval.

* A Cox model adjusting for sex, histological type.

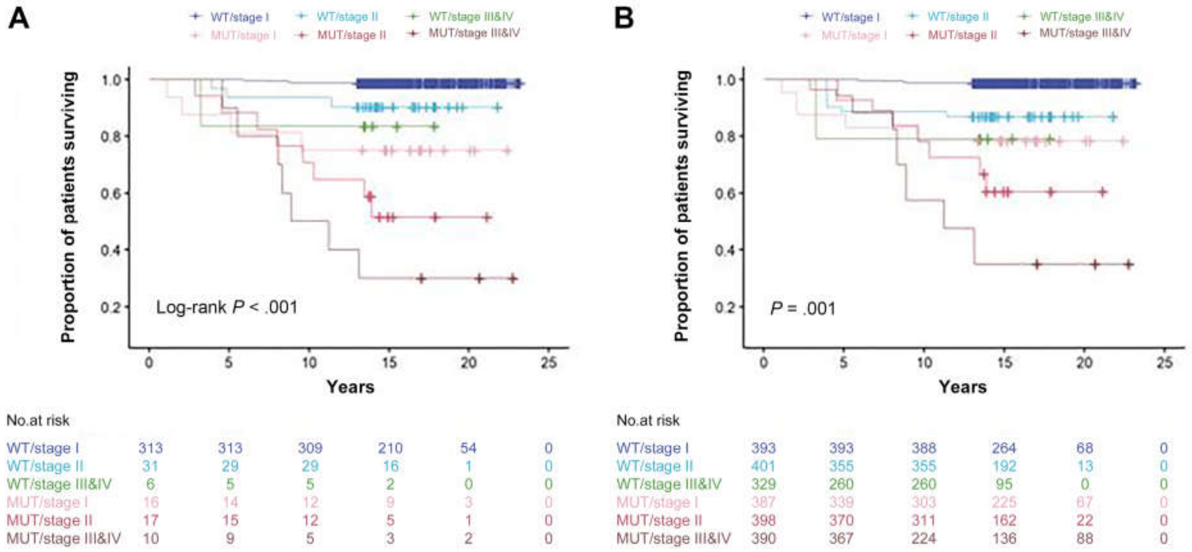

Figure S1. Thyroid cancer-specific survival according to the 6 combinations of TNM-8 stage and *TERT* promoter mutation status with Kaplan-Meier survival curves (A) and with adjusted survival curves (B). .
